# Quantitative analysis of the lysine acetylome reveals the role of SIRT3-mediated HSP60 deacetylation in suppressing intracellular *Mycobacterium tuberculosis* survival

**DOI:** 10.1128/spectrum.00749-24

**Published:** 2024-06-25

**Authors:** Chuanzhi Zhu, Yuheng Duan, Jing Dong, Hongyan Jia, Lanyue Zhang, Aiying Xing, Zihui Li, Boping Du, Qi Sun, Yinxia Huang, Zongde Zhang, Liping Pan

**Affiliations:** 1 Laboratory of Molecular Biology, Beijing Key Laboratory for Drug Resistance Tuberculosis Research, Beijing Tuberculosis and Thoracic Tumor Research Institute, Beijing Chest Hospital, Capital Medical University, Beijing, China; Shenzhen University School of Medicine, Shenzhen, China

**Keywords:** *Mycobacterium tuberculosis*, acetylome, heat shock proteins 60, sirtuin 3, apoptosis

## Abstract

Protein acetylation and deacetylation are key epigenetic modifications that regulate the initiation and development of several diseases. In the context of infection with *Mycobacterium tuberculosis* (*M. tb*), these processes are essential for host–pathogen interactions and immune responses. However, the specific effects of acetylation and deacetylation on cellular functions during *M. tb* infection are not fully understood. This study employed Tandem Mass Tag (TMT) labeling for quantitative proteomic profiling to examine the acetylproteome (acetylome) profiles of noninfected and *M. tb*-infected macrophages. We identified 715 acetylated peptides from 1,072 proteins and quantified 544 lysine acetylation sites (Kac) in 402 proteins in noninfected and *M. tb*-infected macrophages. Our research revealed a link between acetylation events and metabolic changes during *M. tb* infection. Notably, the deacetylation of heat shock protein 60 (HSP60), a key chaperone protein, was significantly associated with this process. Specifically, the deacetylation of HSP60 at K96 by sirtuin3 (SIRT3) enhances macrophage apoptosis, leading to the elimination of intracellular *M. tb*. These findings underscore the pivotal role of the SIRT3–HSP60 axis in the host immune response to *M. tb*. This study offers a new perspective on host protein acetylation and suggests that targeting host-directed therapies could be a promising approach for tuberculosis immunotherapy.

**IMPORTANCE:**

Protein acetylation is crucial for the onset, development, and outcome of tuberculosis (TB). Our study comprehensively investigated the dynamics of lysine acetylation during *M. tb* infection, shedding light on the intricate host–pathogen interactions that underlie the pathogenesis of tuberculosis. Using an advanced quantitative lysine proteomics approach, different profiles of acetylation sites and proteins in macrophages infected with *M. tb* were identified. Functional enrichment and protein–protein network analyses revealed significant associations between acetylated proteins and key cellular pathways, highlighting their critical role in the host response to *M. tb* infection. Furthermore, the deacetylation of HSP60 and its influence on macrophage-mediated clearance of *M. tb* underscore the functional significance of acetylation in tuberculosis pathogenesis. In conclusion, this study provides valuable insights into the regulatory mechanisms governing host immune responses to *M. tb* infection and offers promising avenues for developing novel therapeutic interventions against TB.

## INTRODUCTION

Tuberculosis (TB) continues to pose a significant global health challenge, affecting millions of individuals annually. *Mycobacterium tuberculosis (M. tb),* the causative agent of TB, has evolved intricate mechanisms to evade host immune responses and establish persistent infections (1). Understanding the molecular mechanisms that govern the interplay between the host and pathogen is essential for devising effective strategies to combat TB. The discovery of host factors subverted by *M. tb* has yielded crucial insights into the pathogenesis of TB and paved the way for the development of host-directed therapy (HDT) (2). Recent advancements in HDT have offered promising strategies for combating TB (3).

Post-translational protein modifications (PTMs) are pivotal in modulating the immune response of macrophages to *M. tb* infection (4). Among these PTMs, acetylation, the addition of an acetyl group to specific amino acid residues, is a critical regulator of protein function, stability, and protein–protein interactions (5). Lysine acetylation (Kac) has emerged as a key player in modulating host immunity against intracellular bacterial survival. By modulating the acetylation status of specific proteins, pathogens, such as *M. tb*, can manipulate host cell signaling pathways and evade immune defenses (6). This targeted modulation of Kac by pathogens underscores the importance of understanding the acetylation landscape during infection. We can gain valuable insights into the mechanisms underlying immune regulation and disease pathogenesis by deciphering the intricate interplay between Kac and host–pathogen interactions.

Acetylation of both histone and non-histone proteins plays a crucial role in TB pathogenesis and the host immune response. *M. tb* modulates histone acetylation patterns, affecting gene expression in immune responses. For instance, *M. tb* inhibits histone H3 acetylation in the interleukin-12B (IL-12B) promoter region, downregulating IL-12B expression and suppressing the Th1 immune response, which promotes *M. tb* survival (7). Additionally, *M. tb* suppresses human leukocyte antigen DR (HLA-DR) gene expression by recruiting the histone deacetylase (HDAC) complex to its promoter, facilitating intracellular survival (8). In TB patients, the expression of H3K14Ac, a specific histone acetylation marker, is reduced in peripheral blood lymphocytes. This reduction is particularly pronounced in the promoter regions of TNF-α and IL-12B, two key cytokines involved in the immune response against *M. tb*. Interestingly, the extent of H3K14Ac reduction in these promoter regions correlates with the survival rate of TB patients (9). Furthermore, *M. tb* manipulates histone acetylation to regulate the expression of matrix metalloproteinases (MMP1 and MMP3). MMPs are enzymes that play a crucial role in TB immune pathogenesis by facilitating bacterial invasion and tissue destruction. *M. tb* modulates MMP expression through the activity of histone deacetylases (HDACs) and histone acetyltransferases (HATs), highlighting the importance of histone acetylation in TB pathogenesis (10). Histone acetylome-wide analyses revealed differential acetylation sites in various cell types of TB patients, revealing that *M. tb* regulates H3K27Ac in the KCNJ15 promoter, promoting apoptosis and bacterial clearance (11). Additionally, non-histone acetylation influences autophagy, apoptosis, and innate immune responses during *M. tb* infection. Non-histone acetylation also influences TB pathogenesis. SIRT1 activation during *M. tb* infection triggers autophagy by deacetylating MAP1LC3B/LC3B, restricting *M. tb* growth (12). Despite progress in understanding the impact of acetylation on host immune responses and pathogen survival, the lysine acetylproteome (acetylome) of host proteins and its function in *M. tb*-infected macrophages remain largely unknown.

The quantitative analysis of lysine acetylomes is a robust methodology that allows for the comprehensive examination of protein acetylation levels (13). This technique leverages mass spectrometry-based approaches to detect and quantify acetylated peptides in a high-throughput fashion. By comparing the acetylome profiles of *M. tb*-infected and uninfected macrophages, we identified specific alterations in protein acetylation linked to *M. tb* infection. Understanding the acetylation patterns of host proteins influenced by *M. tb* infection has the potential to unveil critical molecular events that support bacterial survival and persistence within macrophages (14). Targeting pathways associated with the acetylation of non-histone proteins could offer promising avenues for host-directed therapy against TB.

This study employed TMT labeling for quantitative proteomic profiling to examine the acetylproteome (acetylome) profiles of noninfected and *M. tb*-infected macrophages. We identified 715 acetylated peptides from 1072 proteins and quantified 544 lysine acetylation sites (Kac) in 402 proteins in noninfected and *M. tb*-infected macrophages. Our research revealed a link between acetylation events and metabolic changes during *M. tb* infection. This finding highlights the relationships among *M. tb* infection, acetylation, and macrophage metabolic reprogramming. The deacetylation of HSP60, a key chaperone protein, was significantly associated with this process. Specifically, the deacetylation of HSP60 at K96 by SIRT3 enhances macrophage apoptosis, eliminating intracellular *M. tb*. These findings underscore the pivotal role of the SIRT3–HSP60 axis in the host immune response to *M. tb*. This study offers a new perspective on host protein acetylation and suggests that targeting host-directed therapies could be a promising approach for tuberculosis immunotherapy.

## MATERIALS AND METHODS

### Bacteria and cells

The *M. tb* (H37Ra and H37Rv) strains were previously preserved in our laboratory. *M. tb* was grown in Difco Middlebrook 7H9 broth supplemented with 10% oleic acid–albumin–dextrose–catalase (OADC) enrichment (BD, USA), 0.05% (vol/vol) Tween 80 (Sigma, USA), and 0.2% (vol/vol) glycerol (Thermo Fisher, USA) at 37°C.

The THP-1 cell line was obtained from the Cell Bank of the Chinese Academy of Sciences (Shanghai, China), and HEK 293T cells were preserved in our laboratory. The cells were cultured in RPMI 1640 medium or Dulbecco's Modified Eagle's Medium (DMEM) supplemented with 10% fetal bovine serum (FBS) (Gibco, USA) in a CO_2_ incubator at 37°C. THP-1 cells were treated with 20 ng/mL phorbol 12-myristate 13-acetate (PMA) (Sigma, USA) for 48 h, followed by a 12-h rest before infection.

### Cell infection for acetylome analysis

Phorbol-12-myristate-13-acetate (PMA)-differentiated macrophages (3 × 10^7^) were cultured in 150-mm plates (Costar, USA). H37Ra was cultured to an OD600 between 0.6 and 0.8, separated into individual strains, and measured using a bacterial ultrasonic dispersion counter (TB Healthcare, China). The three plates of PMA-induced macrophages were infected with H37Ra at a multiplicity of infection of 5 (MOI = 5) at 24 h, whereas the other three plates served as the control group.

### Protein extraction, digestion, and TMT labeling

H37Ra-infected (*n* = 3) and noninfected (*n* = 3) macrophages were collected and sonicated in lysis buffer (8 M urea, 30 mM HEPES, 1 mM Na_3_VO_4_, 2.5 mM Na_3_PO_4_, 5 mM C_4_H_7_NaO_2_). After centrifugation at 20,000 × *g* at 4°C for 30 min, the supernatant was extracted, and dithiothreitol (DTT) (Promega, USA) was added to a final concentration of 10 mM, followed by the addition of iodoacetamide (Promega, USA) to a final concentration of 55 mM. Protein quantification was performed using the Bradford method (Bio-Rad, USA). The equivalent protein samples were treated with 50% triethylammonium bicarbonate (TEAB) (pH 8.0) (Sigma, USA) to remove urea through the use of a 10-K ultrafiltration tube at 14,000 × *g* at 4°C for 40 min. Trypsin (Promega, USA) was added at a 1:30 trypsin-to-protein mass ratio at 37°C overnight digestion. After the digestion solution was freeze–dried, the peptide segments were resolved using 30 µL/tube TEAB (water: TEAB = 1:1). The peptides were labeled using a TMT10-plex isobaric label reagent set kit (Thermo Scientific, USA) according to the manufacturer’s protocol. The *M. tb*-infected and noninfected samples were labeled with the TMT reagents 126, 127 N, 128C, 129 N, 129C, and 131. The peptide mixtures with different TMT reagents were incubated for 1  h at room temperature, and then 5% hydroxylamine (Thermo Scientific, USA) was added for 15 min at room temperature. The labeled samples were dried by vacuum centrifugation.

### Affinity enrichment and high performance liquid chromatography (HPLC) fractionation

The peptide segment was dissolved in immunoaffinity purification (IAP) buffer (50 mM 4-Morpholinepropanesulfonic acid pH 7.2, 10 mM Na_3_PO_4_, 50 mM NaCl) to a final concentration of 1 µg/mL and enriched using lysine acetylation antibody binding beads (IMMUNECHEM, China). The samples and beads were mixed overnight at 4°C with rotary shaking. After centrifugation, the beads were washed twice with 1 mL of IAP buffer and thrice with cold Milli-Q water. The peptide segment was washed twice with 55 µL of 0.15% TFA at room temperature for 10 min and dissolved in 0.1% FA. Each peptide segment sample was then separated by HPLC using C18 small columns (Phenomenex). The sample was eluted with 10%, 15%, 18%, 25%, and 50% acetonitrile, and the above components were collected separately and dried under vacuum.

### Liquid chromatography tandem mass spectrometry (LC-MS/MS) detection and analysis

The five preseparated peptides were detected by a Q Exactive mass spectrometer (Thermo Scientific, USA) coupled to a Dionex Ultimate 3000 nano-LC system. The peptides were separated using a reverse‐phase analytical column (NanoLC trap, Thermo Scientific, USA). The gradient was a linear increase from 5% to 30% solvent B (0.1% FA in 98% ACN) over 30 min, from 30% to 60% for 5 min, from a linear increase to 80% over 3 min, and then maintained at 80% for the last 7 min. The flow rate was constant at 0.4 µL/min.

### Database searches using Mascot

After mass spectrometry scanning, the original mass spectrometry files were obtained. These files were input into Proteome Discoverer (PD 1.4) software, which screens mass spectrometry spectra. The spectra extracted by PD were searched using Mascot version 2.3.0. The parameters for the database search included the following: enzyme, trypsin; fixed modification, carbamidomethyl (C); and variable modifications, oxidation (M), deamination (N-term Q), TMT six plex (K), acetyl (N-term), and acetyl (K). The database number used was 2019-UniProt-human 9606. A database search was performed, and the results were filtered based on a false discovery rate (FDR) < 1% to obtain peptide and protein identification results. Subsequently, the acetylated sites were further filtered at a site decoy fraction ≤1% to identify significant modifications. For the identification and quantification of proteins, the *P* value was set at ≤0.05, and acetylated proteins with a fold change of upregulation ≥1.25 or downregulation ≤0.75 were considered differentially acetylated proteins between the *M. tb*-infected and uninfected groups. Additionally, among the three sets of replicates, at least one set met the difference condition, with a minimum of two-thirds or more of the comparative differences aligning in the same direction as the specified difference condition.

### Acetylated protein annotation

Gene Ontology (GO) and Kyoto Encyclopedia of Genes and Genomes (KEGG) databases were used for enrichment analysis of differentially acetylated proteins. Significantly enriched terms had a threshold of *P* < 0.01 for GO analysis and *P* < 0.05 for KEGG pathway analysis. The GO terms were further divided into three subgroups: biological process (BP), cellular component (CC), and molecular function (MF). Furthermore, protein–protein interaction (PPI) network analysis was performed using the Search Tool for the Retrieval of Interacting Genes (STRING) database and Cytoscape.

### Plasmid and lentiviral construction

The mRNA from THP-1 macrophages and cDNA clones encoding HSP60 were subcloned and inserted into the pLvx-Flag vector from Xu’s laboratory (Shenzhen University). The pLvx-HSP60 plasmid was used as a template for site-directed mutagenesis via the Mut Express II Fast Mutagenesis Kit V2 (Vazyme, China). The primers K96R were used for site-directed mutagenesis to replace AAA (K96) with AGA (R96) for pLvx-HSP60^K96R^ and AAA (K96) with CAA (Q96) for pLvx-HSP60^K96Q^. The primers used were as follows: *HSP60*-F: 5′-GATGACGATGACAAGCTCGAGATGCTTCGGTTACCCACAGTCT-3′, *HSP60*-R: 5′-CGCGGGCCCTCTAGACTCGAGTTAGAACATGCCACCTCCCATAC-3′, *HSP60*^K96R^-F: 5′-TGGAGCTAGACTTGTTCAAGATGTTGCCAATAACA-3′, *HSP60*^K96R^-R: 5′-GAACAAGTCTAGCTCCAATGTTTTTGTATTTATCTTTT-3′, *HSP60*^K96Q^-F: 5′-TGGAGCTCAACTTGTTCAAGATGTTGCCAATAA-3′, and *HSP60*^K96Q^-R: 5'- GAACAAGTTGAGCTCCAATGTTTTTGTATTTATCTTTT-3′. All the constructs were confirmed by DNA sequencing (Genewiz, China). These lentivirus plasmids (2.5 µg) and packaging plasmids (psPAX2 2.5 µg and pMD2. G 1.5 µg) were transfected into HEK239 cells using FuGENE6 Transfection Reagent (Promega, USA). The lentivirus was collected at 48 h post-transfection.

### RNA interference

The siRNA oligonucleotide duplexes targeting *HSP60* (sequence: GAGGCTATATTTCTCCATA) and the negative control (sequence: UUCUCCGAACGUGUCACGU) were synthesized by Ribo-bio (Ribo-bio, China). A total of 5 × 10^5^ PMA-differentiated macrophages were transfected with siRNA using Lipofectamine RNAiMax (Invitrogen, USA) at a final concentration of 20 nM. At 48 h after transfection, the macrophages were infected with H37Rv (MOI = 5) for 24 h. The supernatants of the cell lysates were subjected to immunoprecipitation (IP) and then analyzed by immunoblotting.

### Inhibitor treatments

A total of 5 × 10^5^ PMA-differentiated macrophages were cultured in 12-well plates and pretreated with the SIRT3 activator honokiol (HKL, 10 µM) or the SIRT3 inhibitor 3-(1H-1,2,3-triazol-4-yl) pyridine (3-TYP, 25 µM) obtained from MedChemExpress (MCE, China) for 16 h before *M. tb* infection.

### IP and immunoblotting

Cell lysates were harvested in lysis buffer (150 mM NaCl, 20 mM Tris-HCl [pH 7.5], 1 mM EDTA, 0.5% Nonidet P-40), and a protease inhibitor cocktail was added (Roche, USA), as previously described (15). Antibodies (2 µg) and control IgG (1 µg) were used for IP and incubated at 4°C overnight. The samples were separated by SDS‒PAGE and transferred onto polyvinylidene fluoride (PVDF) membranes (Merck Millipore, USA). After blocking with 5% skim milk (BD, USA) in phosphate buffer solution with 0.05% Tween-20 (PBST ) for 2 h at room temperature, the membranes were incubated with primary antibodies against the target proteins overnight at 4°C with shaking. After washing thrice with PBST, the membranes were incubated with peroxidase-conjugated secondary antibodies for 1.5 h at room temperature and visualized using enhanced chemiluminescence (ECL) detection solution (Thermo Scientific, USA). The following antibodies were used in this study: anti-HSP60 (Proteintech, China), anti-SOD2 and anti-SOD2 K68Ac (Abcam, USA), anti-SIRT3, anti-caspase-3, anti-cleaved caspase-3, and acetylated-lysine (CST, USA), anti-Flag (MBL, Japan), HRP-linked acetyl-lysine (IMMUNECHEM, China), HRP-linked anti-rabbit IgG and anti-mouse IgG (ZSGB-BIO, China).

### Intracellular bacterial survival assay

PMA-differentiated macrophages (6 × 10^5^) overexpressing pLvx-HSP60, pLvx-HSP60^K96Q^, and pLvx-HSP60^K96R^ were infected with H37Rv (MOI = 5) for 6 h, followed by three washes to remove extracellular bacilli. Subsequently, the infected macrophages were treated with HKL or 3-TYP for 72 h. Intracellular bacterial levels were assessed after a 10-fold dilution at 72 h after infection.

### Statistical analysis

The data were statistically analyzed using GraphPad Prism (version 8.0, CA, USA). The data were presented as the mean ± standard deviation. Student’s *t*-tests were used to compare the two groups. One-way analysis of variance was used to compare the differences among multiple groups with Bonferroni adjustment. Differences were considered significant at *P* < 0.05. The detailed statistical tests used in each analysis were described in the main text and figure legends.

## RESULTS

### Profiles of Kac sites and proteins in *M. tb*-infected and uninfected macrophages

**Fig 1:**
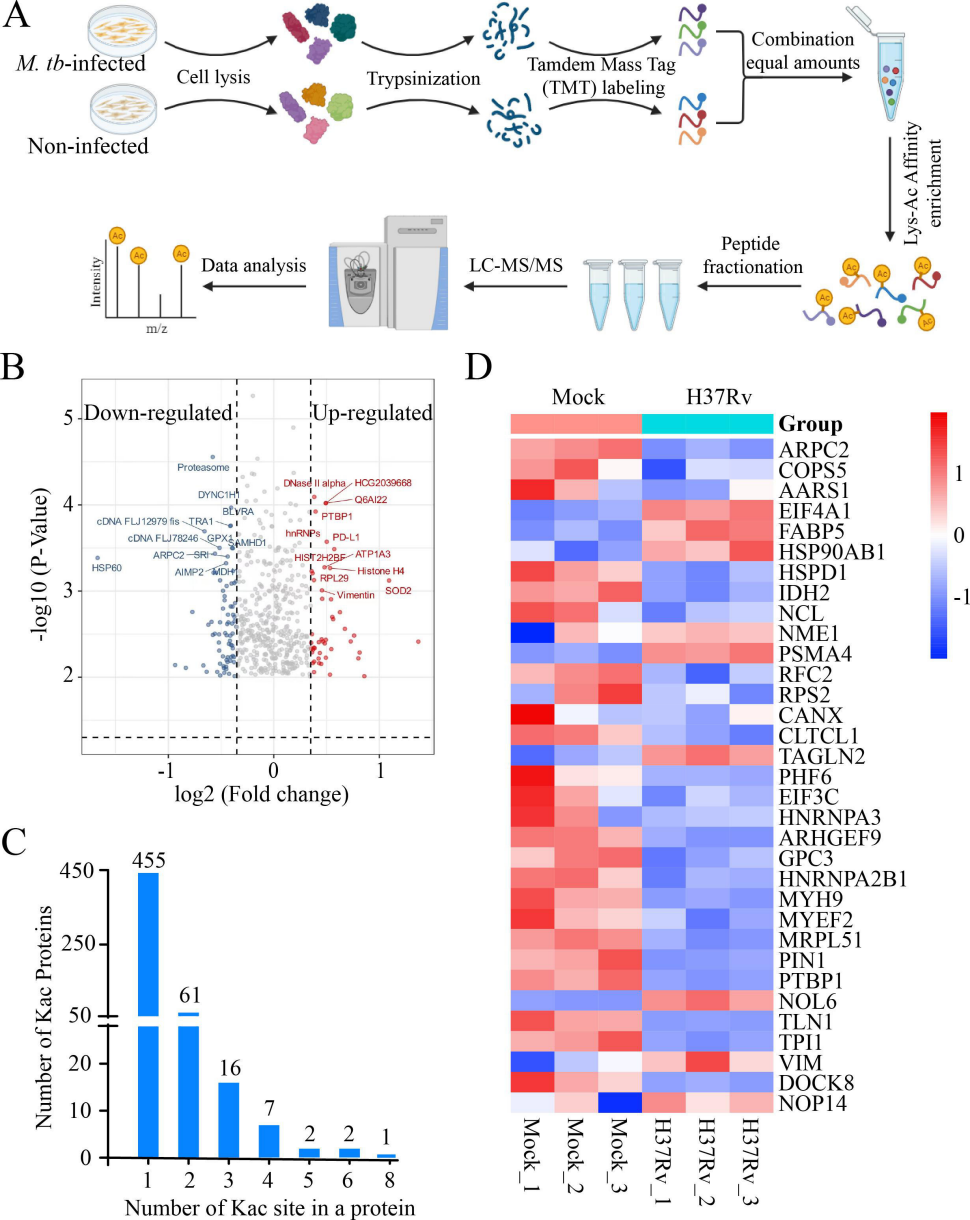
Acetylome analysis in *M. tb*-infected macrophages. (**A**) Tandem mass tag (TMT) labeling strategy for quantifying the acetylome in non- and *M. tb*-infected macrophages. (**B**) Differentially acetylated proteins in *M. tb*-infected macrophages compared with uninfected groups. (**C**) Distribution of identified acetylated sites on proteins. (**D**) Transcript expression levels of Kac proteins in non- and *M. tb*-infected macrophages.

To explore the acetylation landscape of host proteins during *M. tb* infection, we conducted a comprehensive quantitative acetylome analysis using a TMT labeling-based quantitative proteomics approach (Fig. 1A). A total of 715 acetylation peptides (containing Kac and N-terminal acetyl groups) were identified from the 1,072 quantified proteins (Table S1) between the uninfected and *M. tb*-infected groups. A total of 544 Kac sites in 402 Kac proteins were identified from the 715 acetylation peptides (Table S2). Among the 715 acetylation peptides identified, 146 were unique to the infected group. Compared with those in the noninfected group, 87 peptides from 74 proteins were differentially acetylated in *M. tb*-infected macrophages. The most significantly differentially acetylated proteins were ZF850 and SOD2. Conversely, 59 peptides among the 53 proteins were deacetylated in *M. tb*-infected macrophages compared with those in noninfected macrophages, among which the top three proteins were HSP60, CAP1, and IDH2 (Fig. 1B; Table S3). Notably, most Kac-modified proteins contained one or two lysine residues, and a subset of 28 Kac-modified proteins contained three or more Kac sites (Fig. 1C). This diversity in Kac sites underscores the complexity of the acetylation landscape during *M. tb* infection. By analyzing our previously published transcriptome data set, we identified 47 protein-coding genes with Kac sites that displayed differential expression patterns between noninfected and *M. tb*-infected macrophages. Notably, most of these proteins were not detected through mRNA profiling alone (Fig. 1D; Table S4). Taken together, these results indicate that differential Kac proteins may be associated with infection progression during *M. tb* infection. The integration of acetylome and transcriptome data provides a more comprehensive understanding of regulatory networks during *M. tb* infection.

### Functional enrichment and protein‒protein network analysis of acetylated proteins

**Fig 2:**
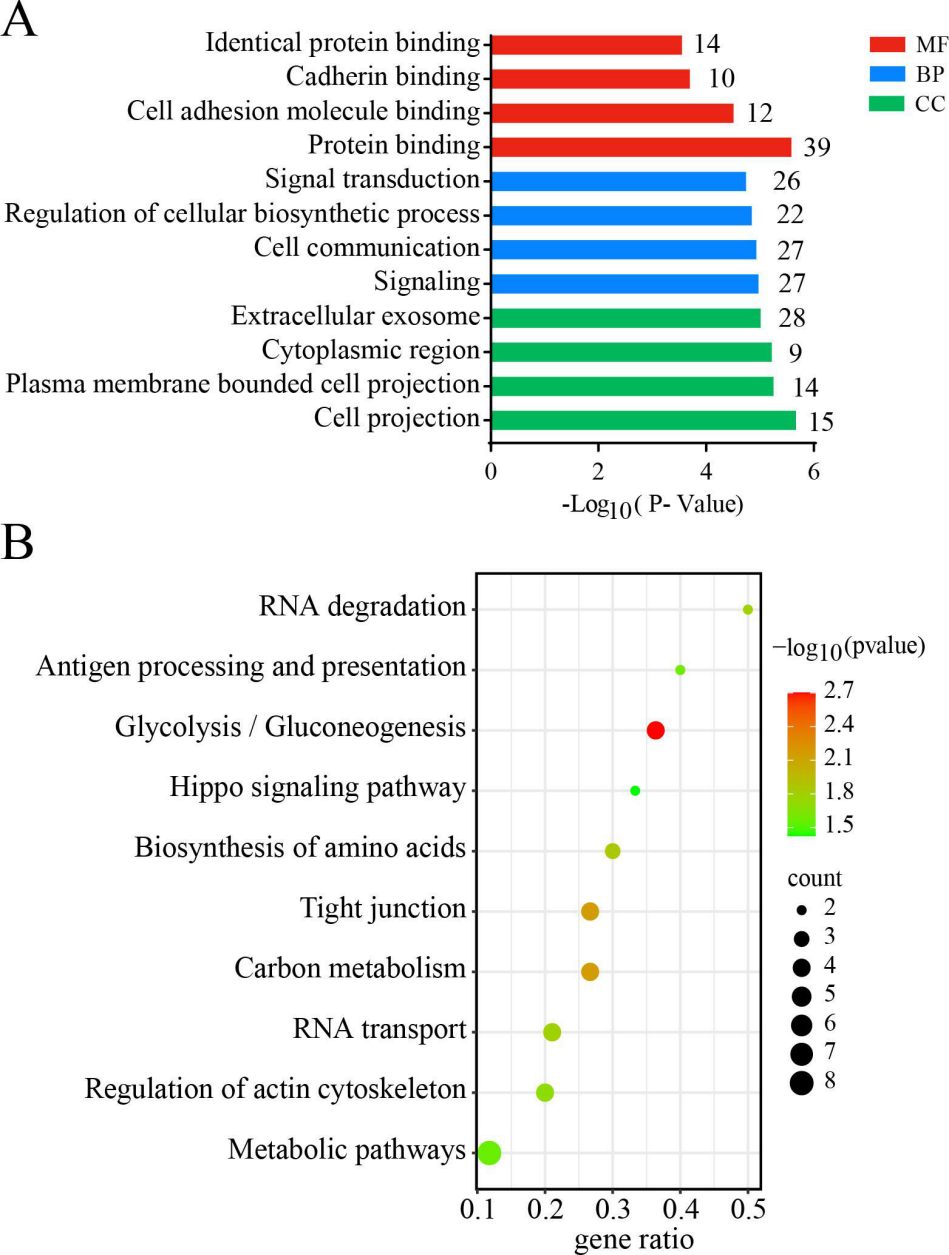
Functional enrichment of differential Kac proteins. (**A**) GO enrichment analysis and (**B**) KEGG pathway analyses of differential Kac proteins in non- and *M. tb*-infected macrophages.

To gain a comprehensive understanding of the functions and cellular distribution of the 127 identified Kac proteins, we conducted enrichment analyses across various biological domains, encompassing GO and KEGG pathway enrichment. BP enrichment analysis revealed that these Kac proteins were enriched in “signaling,” “cell communication,” and “regulation of cellular biosynthetic process,” among others. Molecular function (MF) analysis revealed that these Kac proteins were associated with “protein binding,” “cell adhesion molecule binding,” “cadherin binding,” and “identical protein binding,” among others. According to the Cellular Component (CC) analysis, the Kac proteins were predominantly localized to cellular structures such as “cell projection,” “plasma membrane-bound cell projection,” and “cytoplasmic region” (Fig. 2A; Table S5). Moreover, KEGG pathway analysis revealed significant associations between these Kac proteins and signaling and metabolic pathways, including “metabolic pathways,” “glycolysis/gluconeogenesis,” and “carbon metabolism” (Fig. 2B; Table S5).

**Fig 3:**
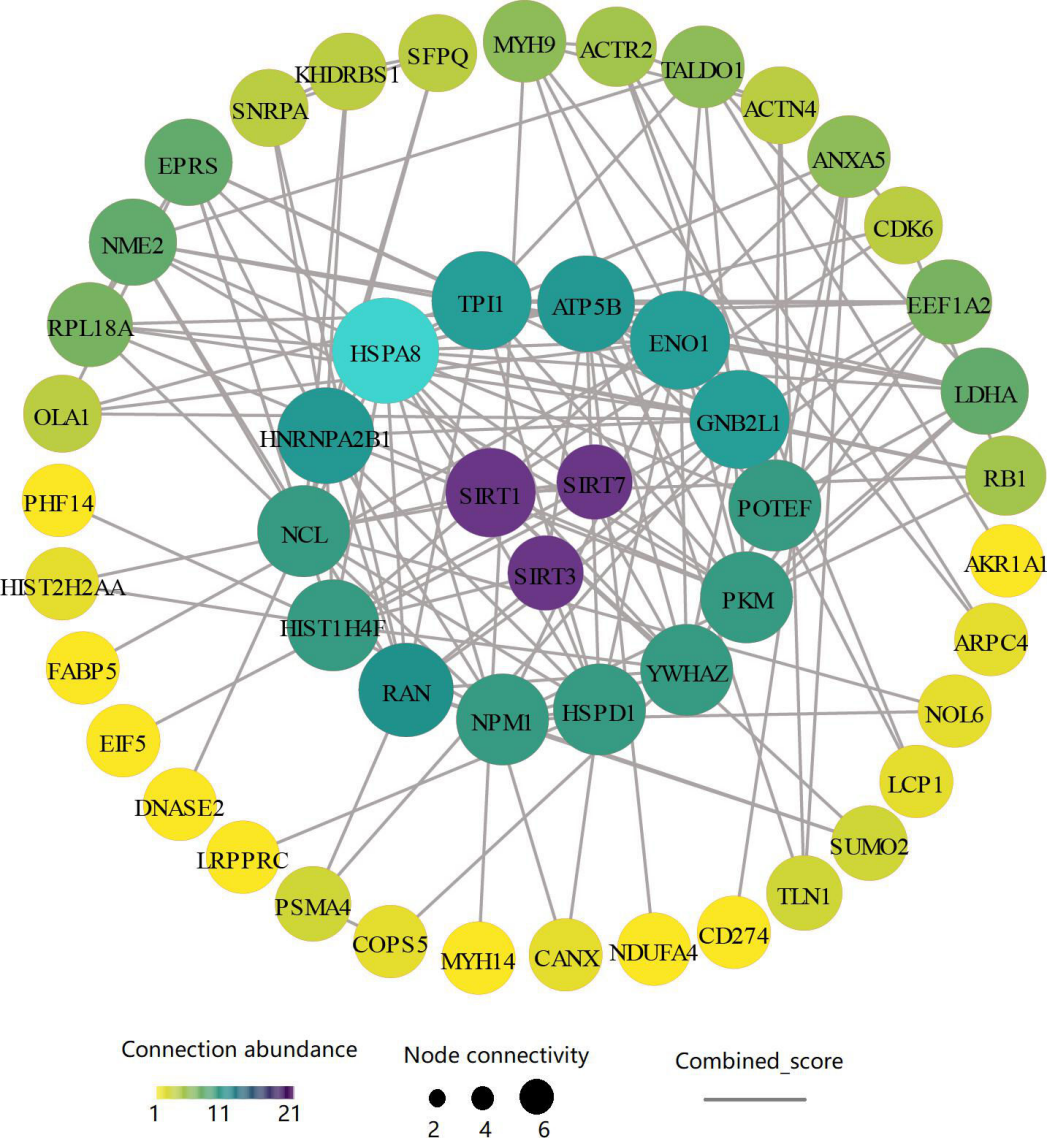
SIRT network and interacting Kac proteins. Sub-network of SIRT and its interacting Kac proteins. Node size represents SIRT’s degree of interaction, whereas color and edge size indicate clustering coefficient and interaction strength, respectively.

Additionally, recognizing the importance of protein‒protein interactions (PPIs) in various biological processes, such as signal transduction and metabolism, we explored PPI networks using the STRING database. This analysis revealed key interactions, including those involving the histone deacetylases SIRT1, SIRT3, and SIRT7, which play critical roles in the killing of intercellular *M. tb* by macrophages via multiple Kac proteins. The results revealed that SIRT interacts with multiple Kac proteins (Fig. 3). In particular, SIRT3 strongly interacts with multiple deregulated Kac proteins, such as HSPD1 (HSP60), LDHA, and ATP5B (Fig. 3). These findings underscore the critical role played by differential Kac proteins regulated by acetyltransferases and deacetylases in modulating signaling and metabolic processes in *M. tb*-infected macrophages.

### Kac protein validation in *M. tb-*infected macrophages

**Fig 4:**
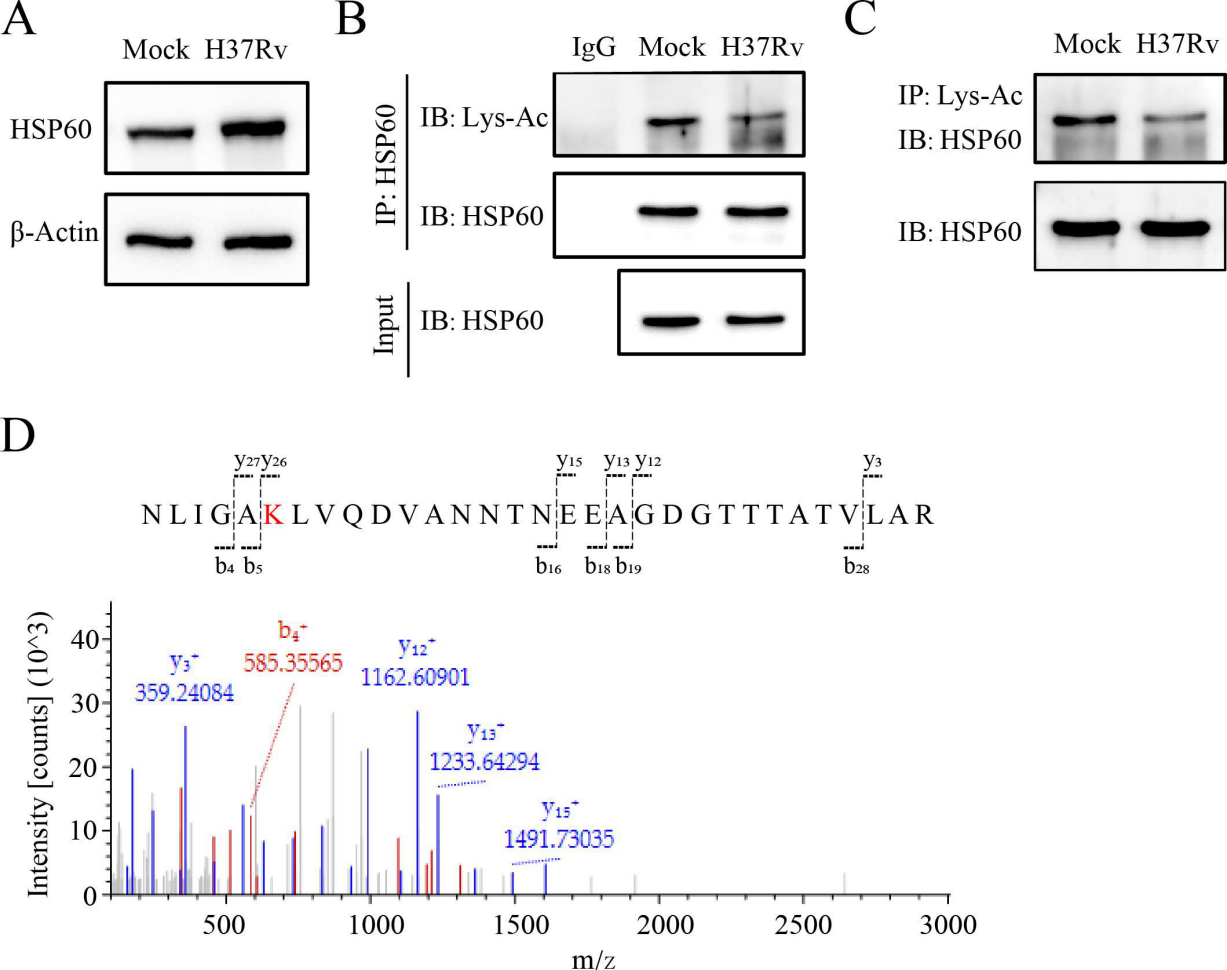
Validation of Kac proteins in *M. tb*- infected macrophages. (**A**) Expression of HSP60 in non- and *M. tb*-infected macrophages. (**B, C**) Acetylation of HSP60 detected using HSP60 antibody and lysine acetylation antibody in non- and *M. tb*-infected macrophages. (**D**) Mass spectroscopy analysis identifying K96 acetylation of HSP60.

As alterations in protein abundance can impact the observed changes in protein acetylation (16, 17), we selected proteins for subsequent acetylation validation based on the analysis of acetylome and transcriptomics data. First, we focused on identifying proteins for which alterations in abundance contradicted changes in acetylation levels, such as those of HSP60. According to the mass spectrometry results, our proteomic analysis revealed six lysine residues (K58, K72, K96, K236, K301, and K387) on HSP60 (Tables S1 and S2), including the most significantly deacetylated lysine site (K96) in *M. tb*-infected macrophages. However, the mRNA expression of HSP60 was greater in *M. tb*-infected macrophages than in uninfected macrophages. Therefore, to validate the changes in the acetylation of HSP60, we first examined the protein expression and acetylation level of HSP60 using Western blotting and IP. Our results showed that HSP60 expression was substantially greater in *M. tb*-infected macrophages than in uninfected macrophages (Fig. 4A). Additionally, the degree of HSP60 acetylation was significantly lower in *M. tb*-infected macrophages than in uninfected macrophages, as evidenced by the results of immunoprecipitation with an anti-HSP60 antibody and subsequent detection with an acetyl lysine antibody (Fig. 4B). This result was further validated using an acetylated-lysine antibody for co-IP and detected using an HSP60 antibody (Fig. 4C). Notably, the acetylation of K96 in HSP60 was detected using LC-MS/MS (Fig. 4D). These results underscore the precision and importance of our quantitative acetylome analysis, particularly in the context of HSP60 acetylation. Thus, we further demonstrated the accuracy of the identified acetylome data and speculated that the deacetylation of HSP60 may be related to the host antimycobacterial immune response.

### HSP60 decreased the intracellular survival of *M. tb* and increased the apoptosis of *M. tb*-infected macrophages

**Fig 5:**
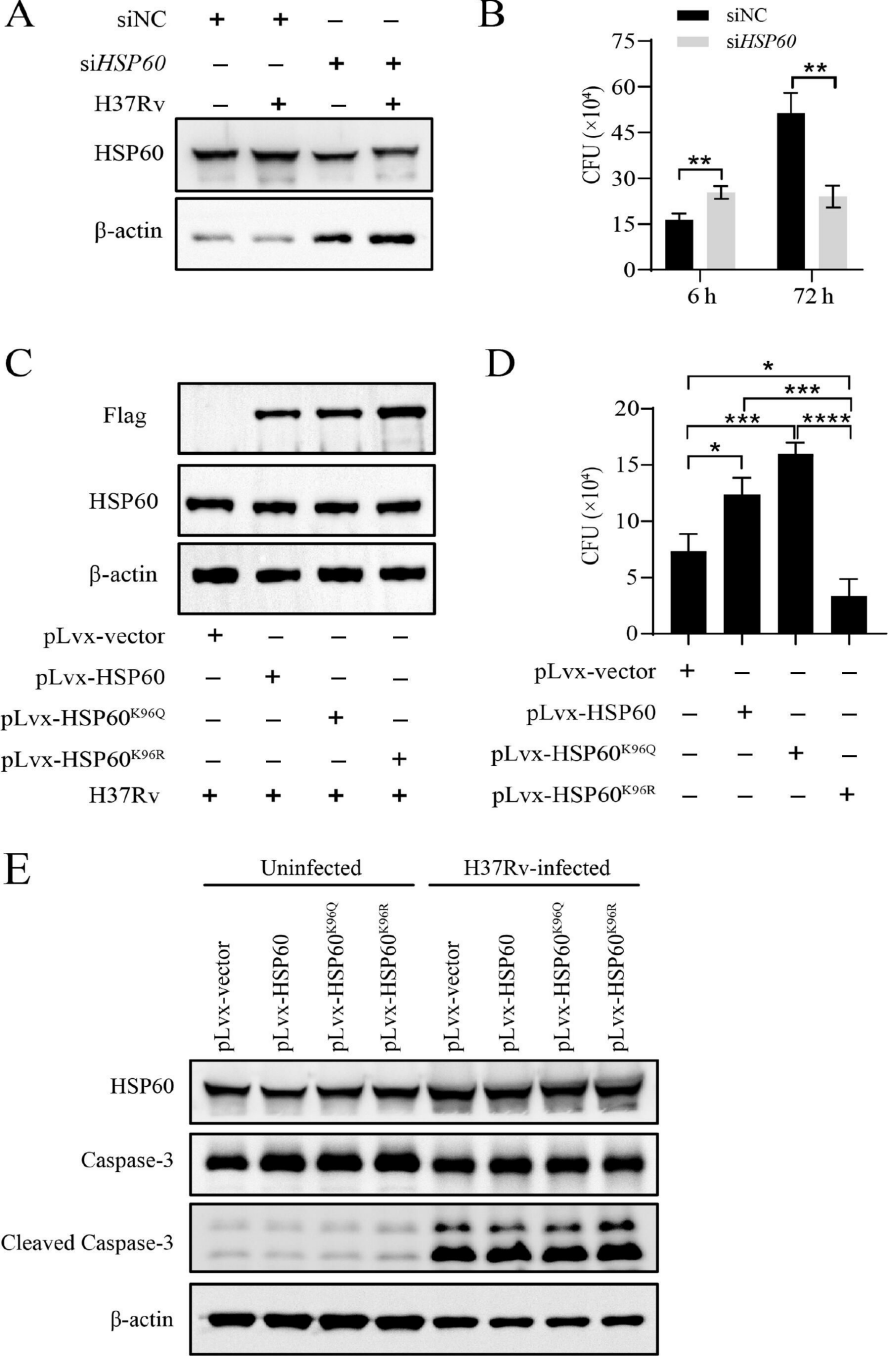
HSP60 deacetylation against intracellular survival of *M. tb* and effect apoptosis in macrophages. (**A**) Levels of HSP60 in macrophages transfected with *HSP60* siRNA for 48 h and then infected with H37Rv for 24 h. (**B**) Bactericidal activity of *HSP60* knockdown macrophages, as detected by CFU counts at 6 h and 72 h post-infection. (**C**) Overexpression of HSP60 and K96 mutation HSP60 proteins in H37Rv-infected macrophages at 24 h. (**D**) Bactericidal activity of HSP60 and K96 mutation HSP60 overexpression macrophages, as detected by CFU counts at 72 h post-infection of *M. tb*. (**E**) The expression of HSP60, cleaved caspase-3, and caspase-3 was assessed at 24 h in macrophages infected with *M. tb* after lentivirus post-infection at 48 h. *P < 0.05, **P < 0.01, ***P < 0.001, ****P < 0.0001.

To investigate the effect of HSP60 and its deacetylation on the intracellular survival of *M. tb*, we evaluated the intracellular viability of *M. tb* in macrophages with HSP60 knockdown, HSP60 overexpression, and point mutations. Our findings confirmed the efficient knockdown of *HSP60* in macrophages using siRNA (Fig. 5A). Remarkably, the intracellular growth of *M. tb* was significantly reduced in *HSP60* knockdown macrophages (Fig. 5B). Conversely, we observed a substantial increase in *M. tb* survival in HSP60-overexpressing macrophages. In particular, the intracellular bacterial load of *M. tb* was similarly increased in macrophages harboring the HSP60^K96Q^ mutation. However, this bacterial load was significantly lower in the HSP60^K96R^-overexpressing macrophages than in the HSP60- and HSP60^K96Q^-overexpressing macrophages (Fig. 5C and D). These results suggested that the high expression of HSP60 induced by *M. tb* infection is detrimental to macrophage resistance to infection. However, HSP60 deacetylation at the K96 site is crucial for the efficient elimination of intracellular *M. tb* by macrophages.

According to previous reports, HSP60 is a dual‐directional regulator of apoptosis in response to extracellular or intracellular stresses (18). Although apoptosis is critical for the elimination of mycobacteria by macrophages, virulent mycobacteria prevent macrophage apoptosis for persistent survival (19). To determine whether HSP60 acetylation plays irreplaceable roles in apoptosis, we measured caspase activation, which plays critical roles in apoptosis (20), in *M. tb*-infected macrophages following the overexpression of HSP60, HSP60^K96Q^, and HSP60^K96R^. Western blotting showed that the levels of the cleaved form of caspase-3 were significantly greater in HSP60^K96R^-overexpressing macrophages after *M. tb* infection than in those in the vector, HSP60 and HSP60^K96Q^ groups. Furthermore, *M. tb*-mediated apoptosis was significantly inhibited in the HSP60- and HSP60^K96Q^-overexpressing macrophages (Fig. 5E), indicating that HSP60K96 deacetylation induces caspase-dependent apoptosis. Together, these findings suggest that the deacetylation of HSP60 at K96 may enhance the clearance of intracellular *M. tb* by inducing apoptosis during infection.

### SIRT3 interacted with HSP60 and mediated its deacetylation to enhance macrophage clearance of intracellular *M. tb*

**Fig 6:**
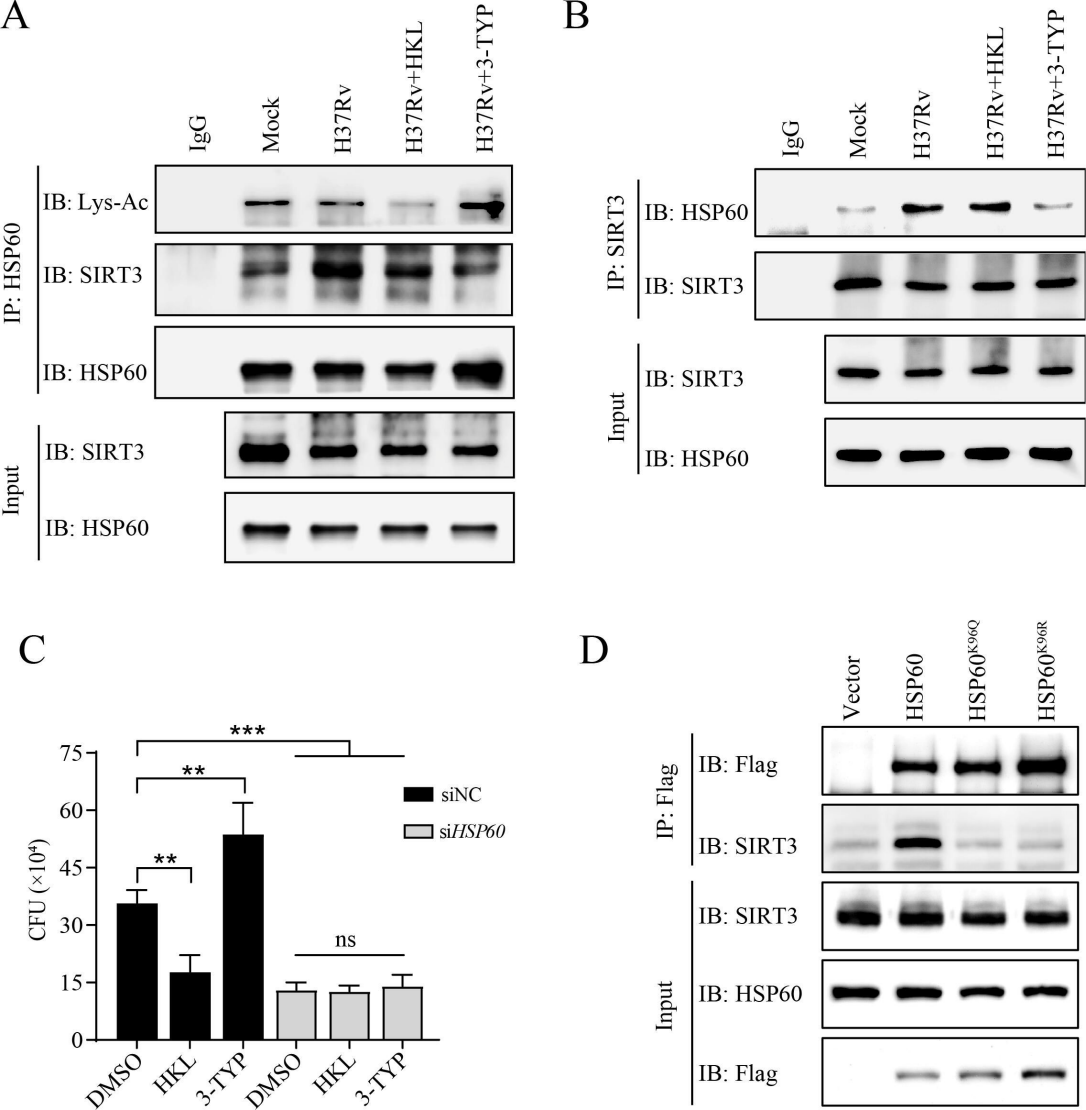
SIRT3-mediated HSP60 deacetylation in *M. tb*-infected macrophages. (**A**) Western blotting and anti-HSP60 IP analysis of non- and *M. tb*-infected macrophages with or without of HKL (10 µM) or 3-TYP (25 µM) treatment. (**B**) Western blotting and anti-SIRT3 IP analysis of *M. tb*-infected macrophages at the percent of HKL and 3-TYP. (**C**) The CFU counts detected at 72 h post-infection of *M. tb* in HSP60 knockdown macrophages following the above treatments. (**D**) Western blotting and anti-Flag IP analysis of HEK293T cells transfected with HSP60, HSP60^K96Q^, or HSP60^K96R^. *P < 0.05, **P < 0.01, ***P < 0.001, ns, not significant.

According to the PPI prediction results, we hypothesized that HSP60 undergoes deacetylation through its interaction with SIRT3 in *M. tb*-infected macrophages (Fig. 3). To substantiate this interaction, we conducted co-IP assays using lysates from both uninfected and *M. tb*-infected macrophages. As shown in Fig. 6A, there was an increase in the association between SIRT3 and HSP60 following *M. tb* infection. Intriguingly, treatment with the SIRT3 inhibitor 3-TYP markedly inhibited this interaction, whereas the SIRT3 agonist HKL had no discernible effect on this interaction during *M. tb* infection (Fig. 6A). We validated the interaction between SIRT3 and HSP60 by employing a SIRT3 antibody for co-IP in *M. tb*-infected macrophages treated with both HKL and 3-TYP (Fig. 6B). Furthermore, HKL treatment significantly attenuated *M. tb*-induced HSP60 deacetylation, whereas 3-TYP significantly increased HSP60 acetylation (Fig. 6A). These findings suggest that inhibiting the deacetylase activity of SIRT3 may impede its interaction with the substrate HSP60 and subsequent acetylation.

As previously reported, SIRT3 is important for the clearance of *M. tb* by macrophages (17, 21). Our results indicated that the killing ability of macrophages was significantly enhanced by HKL treatment and significantly inhibited by the presence of 3-TYP. Importantly, the ability of HSP60 knockdown macrophages to clear intracellular *M. tb* was unaffected by HKL and 3-TYP treatment (Fig. 6C). Additionally, we further explored the role of K96 in HSP60 deacetylation by SIRT3 and conducted experiments involving HEK293T cells transfected with Flag-tagged HSP60, Flag-tagged HSP60^K96Q^, and Flag-tagged HSP60^K96R^ as candidate interaction proteins. The results showed that Flag-tagged HSP60, but not Flag-tagged HSP60^K96Q^ or Flag-tagged HSP60^K96R^, coimmunoprecipitated with SIRT3 (Fig. 6D). These findings collectively suggest that HSP60 is a substrate of SIRT3 and that SIRT3 specifically interacts with HSP60 to mediate the deacetylation of the K96 site. The ability of SIRT3 to enhance macrophage clearance in *M. tb* depends on its interaction with its substrate, HSP60, and promotes its deacetylation.

## DISCUSSION

Lysine acetylation is a post-translational modification that plays a crucial role in the pathogenesis of various diseases, including infectious diseases, cancer, and diabetes (22). Our study investigated the dynamic landscape of acetylated host proteins during *M. tb* infection. We conducted a comprehensive investigation of the acetylome in *M. tb*-infected macrophages to unravel the functional implications of acetylation changes during intracellular infection. By employing advanced proteomics techniques, we identified an extensive catalog of 544 Kac sites within 402 proteins 24 h after infection. Strikingly, we revealed 127 Kac proteins exhibiting differential acetylation levels in *M. tb*-infected macrophages, a significant observation that cannot be solely attributed to changes in mRNA expression levels. Our bioinformatics analysis revealed significant enrichment of metabolic pathways, signal transduction, and immune regulation among the proteins differentially acetylated by *M. tb* infection. This study presents the first comprehensive mapping of the macrophage acetylome during *M. tb* infection, providing valuable insights into the intricate interplay between acetylation dynamics and host–pathogen interactions. Our findings lay the groundwork for further exploration of the role of lysine acetylation in the modulation of cellular processes and immune responses during tuberculosis infection.

The characterization and functional roles of acetylation of histones or non-histone proteins in TB have been outlined for the antimycobacterial immune response (23). Histone acetylation is crucial for regulating gene expression and mediating the immune response during *M. tb* infection. DUSP16/MKP-7 acetylation is essential for autophagy, phagosome maturation, and ROS generation in *M. tb*-infected macrophages (24). The increase in protein acetylation may be a direct result of *M. tb* infection, which impacts protein expression. In contrast, HSP60 expression was upregulated during M. tb infection, whereas its acetylation was significantly reduced in macrophages infected with *M. tb*. This result revealed a substantial decrease in HSP60 acetylation in response to *M. tb* infection, emphasizing the importance of acetylation in shaping the immune landscape. HSP60 is an essential molecular chaperone found in the mitochondria of cells (25). It plays a central role within intracellular molecular networks and acts as a connecting agent in intercellular immune networks (26). Increasing evidence supports the participation of human HSP60 in the pathogenesis of various diseases, including cancer, autoimmune disorders, and infectious diseases (27, 28). However, the specific mechanism of HSP60 in the development of TB remains to be explored further. Moreover, the function of HSP60 may be affected by PTMs, such as phosphorylation, O-GlcNAcylation, and acetylation (29). Dysregulation of HSP60 has been associated with various diseases (30, 31). Previous studies revealed that specific amino acid residues in HSP60, such as K87, K91, K130, K133, K389, and K473, were deacetylated in hepatocellular carcinoma liver tissues compared with normal tissues (32). The hyperacetylation of HSP60, which is induced by anticancer treatment in human tumor cells, contributes to cell death (33). Therefore, the modification of acetylation levels of specific amino acids in HSP60 has the potential to drive the progression of disease. Interestingly, our results revealed that K96 deacetylation in HSP60 significantly improves intracellular bacteria elimination in macrophages infected with *M. tb*. These results suggest that the host may feedback inhibit *M. tb* infection-induced upregulation of HSP60 expression by mediating HSP60 deacetylation, leading to inhibited *M. tb* intracellular survival. Furthermore, a previous study demonstrated that HSP60 is a modulator of apoptosis (30). Undoubtedly, macrophage apoptosis plays pivotal roles in both pathogenesis and host defense against *M. tb* (19). Importantly, acetylated HSP60 undergoes ubiquitination and degradation, resulting in the abrogation of its antiapoptotic effect and subsequent tumor cell death (34). However, the specific HSP60 acetylation residues that inhibit apoptosis are unclear. Our findings provide the first evidence that the deacetylation of the Lys96 site plays a crucial role in enhancing the ability of macrophages to eliminate intracellular *M. tb*, possibly by improving *M. tb*-induced apoptosis. Overall, we propose that HSP60 K96 acetylation plays an important role in controlling the occurrence and development of TB through caspase-dependent apoptosis.

Epigenetic modifications are intricately correlated with the progression of TB (35). Sirtuins, key regulators of host–pathogen interactions, play a critical role in maintaining cellular homeostasis, responding to stress, and regulating energy metabolism (36). SIRT1, SIRT3, and SIRT7 have garnered significant attention for their crucial role in controlling cellular processes, particularly those involved in the host response to *M. tb* infection (17, 21, 37–40). Importantly, SIRT3 not only contributes to anti-inflammatory responses and mitochondrial function but also potentially influences cellular metabolism, underscoring its pivotal role in host defense against mycobacteria (17, 21). Our results revealed that SIRT3 enhances the ability of macrophages to clear intracellular *M. tb* through interactions with HSP60 and mediated-HSP60 deacetylation. In addition, our findings illustrate that manipulating SIRT3 activity with the small molecules HKL and 3-TYP substantially impacts the acetylation of HSP60 during *M. tb* infection. This result is consistent with previous reports showing that the acetylation of the substrate protein HSP60 is mediated by SIRT3 (41). The interaction between SIRT3 and HSP60 during *M. tb* infection also occurs in the presence of HKL and 3-TYP. Furthermore, our results showed that K96 serves as a crucial amino acid site for the interaction between SIRT3 and HSP60 and can be considered a key substrate site for SIRT3. The deacetylation of HSP60, particularly at the K96 site, by SIRT3 suggests a potential regulatory axis influencing the intracellular survival of *M. tb* in macrophages. Taken together, these findings reveal a novel interaction between SIRT3 and HSP60 during *M. tb* infection for the first time and reveal a multifaceted relationship that extends beyond conventional immune responses.

Although our study offers important insights, further investigations are necessary for several reasons. Limitations in protein detection, incomplete proteome coverage, and potential confounding factors from altered protein expression highlight the importance of interpreting acetylation changes with caution. Additionally, the narrow focus on specific stages of *M. tb* infection restricts our understanding of dynamic acetylome changes. Future research should aim to address these limitations, creating a more comprehensive basis for understanding host–pathogen interactions.

In conclusion, our study sheds light on the complex acetylome dynamics in *M. tb*-infected macrophages, emphasizing the crucial role of acetylation in modulating host responses to *M. tb* infection. Specifically, the deacetylation of HSP60 and its interaction with SIRT3 have been identified as key factors in enhancing macrophage-mediated killing of intracellular *M. tb*. These findings suggest that the SIRT3–HSP60 axis represents a novel mechanism that can significantly contribute to our understanding of TB pathogenesis and potentially provide new targets for host-directed therapy.

## Data Availability

The mass spectrometry proteomics data have been deposited to the ProteomeXchange Consortium (https://proteomecentral.proteomexchange.org) via the iProX partner repository with the data set identifier PXD048748.

## SUPPLEMENTAL MATERIAL

Table S1 spectrum.00749-24-s0001.csv All identified acetylation peptides.

Table S2 spectrum.00749-24-s0002.csv Kac sites of identified acetylation peptides.

Table S3 spectrum.00749-24-s0003.csv Different acetylation peptides.

Table S4 spectrum.00749-24-s0004.xlsx FPKM of different acetylation proteins.

Table S5 spectrum.00749-24-s0005.csv Functional enrichment.

ASM does not own the copyrights to Supplemental Material that may be linked to, or accessed through, an article. The authors have granted ASM a non-exclusive, world-wide license to publish the Supplemental Material files. Please contact the corresponding author directly for reuse.
